# Research-based clinical deployment of artificial intelligence algorithm for prostate MRI

**DOI:** 10.1007/s00261-025-05014-7

**Published:** 2025-05-26

**Authors:** Stephanie A. Harmon, Jesse Tetreault, Omer Tarik Esengur, Ming Qin, Enis C. Yilmaz, Victor Chang, Dong Yang, Ziyue Xu, Gregg Cohen, Jeff Plum, Testi Sherif, Ron Levin, Alexander Schmidt-Richberg, Scott Thompson, Samuel Coons, Te Chen, Peter L. Choyke, Daguang Xu, Sandeep Gurram, Bradford J. Wood, Peter A. Pinto, Baris Turkbey

**Affiliations:** 1 National Institutes of Health, Bethesda, USA; 2 Nvidia (United States), Santa Clara, USA; 3 Philips (United States), Andover, USA

**Keywords:** Artificial intelligence, Deep learning, Deployment, PACS, Prostate cancer, MRI

## Abstract

**Purpose:**

A critical limitation to deployment and utilization of Artificial Intelligence (AI) algorithms in radiology practice is the actual integration of algorithms directly into the clinical Picture Archiving and Communications Systems (PACS). Here, we sought to integrate an AI-based pipeline for prostate organ and intraprostatic lesion segmentation within a clinical PACS environment to enable point-of-care utilization under a prospective clinical trial scenario.

**Methods:**

A previously trained, publicly available AI model for segmentation of intra-prostatic findings on multiparametric Magnetic Resonance Imaging (mpMRI) was converted into a containerized environment compatible with MONAI Deploy Express. An inference server and dedicated clinical PACS workflow were established within our institution for evaluation of real-time use of the AI algorithm. PACS-based deployment was prospectively evaluated in two phases: first, a consecutive cohort of patients undergoing diagnostic imaging at our institution and second, a consecutive cohort of patients undergoing biopsy based on mpMRI findings. The AI pipeline was executed from within the PACS environment by the radiologist. AI findings were imported into clinical biopsy planning software for target definition. Metrics analyzing deployment success, timing, and detection performance were recorded and summarized.

**Results:**

In phase one, clinical PACS deployment was successfully executed in 57/58 cases and were obtained within one minute of activation (median 33 s [range 21–50 s]). Comparison with expert radiologist annotation demonstrated stable model performance compared to independent validation studies. In phase 2, 40/40 cases were successfully executed via PACS deployment and results were imported for biopsy targeting. Cancer detection rates for prostate cancer were 82.1% for ROI targets detected by both AI and radiologist, 47.8% in targets proposed by AI and accepted by radiologist, and 33.3% in targets identified by the radiologist alone.

**Conclusions:**

Integration of novel AI algorithms requiring multi-parametric input into clinical PACS environment is feasible and model outputs can be used for downstream clinical tasks.

**Supplementary Information:**

The online version contains supplementary material available at 10.1007/s00261-025-05014-7.

## Introduction

The use of multiparametric MRI (mpMRI) has improved the detection and staging of prostate cancer (PCa) compared to conventional techniques. Specifically, the use of MRI-ultrasound fusion-guided biopsies has significantly improved the accuracy of biopsy targeting and risk assessment of PCa patients, with regions-of-interest (ROIs) identified on mpMRI guiding biopsy acquisition as opposed to traditional systematic 12-core ultrasound-guided biopsies [1–3]. However, substantial inter-reader variability in the interpretation of prostate mpMRI has led to inconsistent clinical utility [4, 5]. Artificial intelligence (AI) algorithms have shown great promise in detection of clinically significant findings at mpMRI, whether in reference to radiologist or pathologist standards, summarized in several comprehensive reviews [6–8]. Unfortunately, very few commercial algorithms are available, leaving majority of these promising algorithms at the level of academic exercises. Recent success in application of advanced AI algorithms in radiology necessitates prospective validation in integrated clinical environments to ensure models are robust, generalizable, and clinically useful [9].

Clinical deployment of AI algorithms is critically limited by the lack of integrated systems, complexities and domain-knowledge of computational requirements, and human research and privacy protective regulations for research-based / non-commercial applications [10–13]. For these reasons, utilization or validation of AI-based algorithms is often completed offline, out of the clinical environment, thereby limiting point-of-care assessment. Commercial solutions are recently becoming available, often utilizing cloud-based computing resources to deliver point-of-care solutions available by purchase. However, publicly available or open source solutions can be utilized by individual researchers, hospitals with limited resources and commercial entities alike, representing an ideal solution for validation and deployment of new cutting-edge algorithms [12, 13]. Dedicated inference machines with user-controlled access may enable AI integration within clinical Picture Archiving and Communication systems (PACS) for translational steps or customized clinical research protocols.

The purpose of this study is to demonstrate the feasibility of real-time deployment of a deep learning-based prostate and lesion segmentation pipeline in a clinical PACS environment, piloting its application for biopsy target selection and delineation.

## Methods

### Patient population

This study includes patients undergoing routine multi-parametric (mpMRI) for clinical assessment or suspicion of localized PCa. The study includes two phases: first, deployment of an AI model into the clinical PACS system for an all-comer population and second, deployment and utilization of an AI model into the clinical PACS system and biopsy planning system for patients undergoing MRI-TRUS fusion-guided biopsy. Clinical protocols were approved by an institutional review board (IRB) and all patients provided written informed consent to participate. A single expert genitourinary radiologist interpreted all images within PACS following routine clinical care guidelines. In Phase 1 (prospective deployment in PACS interface) patients enrolled in a clinical protocol assessing radiological profiling of PCa and imaging-based biomarkers (NCT03354416) and undergoing mpMRI from 04/01/2024-04/29/2024 were considered for study inclusion. No exclusions were applied. In Phase 2 (prospective deployment in PACS and biopsy planning interface) patients jointly enrolled in a clinical protocol assessing radiological profiling of PCa and imaging-based biomarkers (NCT03354416) and a clinical protocol assessing the clinical utility of electromagnetic tracking during interventional procedures (NCT00102544) and undergoing mpMRI prior to MRI-TRUS fusion-guided biopsy between 05/28/24 and 07/09/24 were considered for study inclusion. In Phase 2, no exclusions were made for AI deployment; however, for statistical analysis patients were excluded on the basis of prior treatment or cancelled biopsy session.

### Prostate organ and lesion segmentation pipeline

The model utilized for this study was previously developed using 1390 prostate mpMRI scans from two different institutions [14]. Briefly, the pipeline consists of a series of cascaded AI algorithms. Axial T2W, Apparent Diffusion Coefficient (ADC), and high b-value diffusion images (defined as b-value ≥ 1400) are accepted as input. The T2W series is utilized for automated prostate organ segmentation from a deep-learning based model [15], after which all sequences and organ mask are input to deep-learning based lesion segmentation model [14]. The lesion segmentation model is the final product of an ensemble of 5 models produced during 5-fold cross-validation at initial development. The model is initially trained to segment all intraprostatic lesions with PI-RADSv2 scores 2–5, with the radiologist-defined volume as reference standard. The model achieved Sorenson-Dice Coefficient (DSC) of 0.359 with 56.1% sensitivity compared to expert radiologist during initial testing and has been validated in several studies thereafter with respect to cancer detection [16], an external center [17], external MRIs referred to our center [18], association to radical prostatectomy histology [19], post-focal therapy [20], and post-radiotherapy [21]. This pipeline was trained using NVIDIA Clara Train SDK [22] and was subsequently converted for use in MONAI, an open-source AI software framework for accelerating research and clinical collaboration in medical imaging [23]. The models have been made publicly available for research use https://github.com/Project-MONAI/research-contributions/tree/main/prostate-mri-lesion-seg. The final outputs of the model pipeline include a prostate organ segmentation mask and prostate lesion segmentation mask.

### Deployment framework

**Fig. 1:**
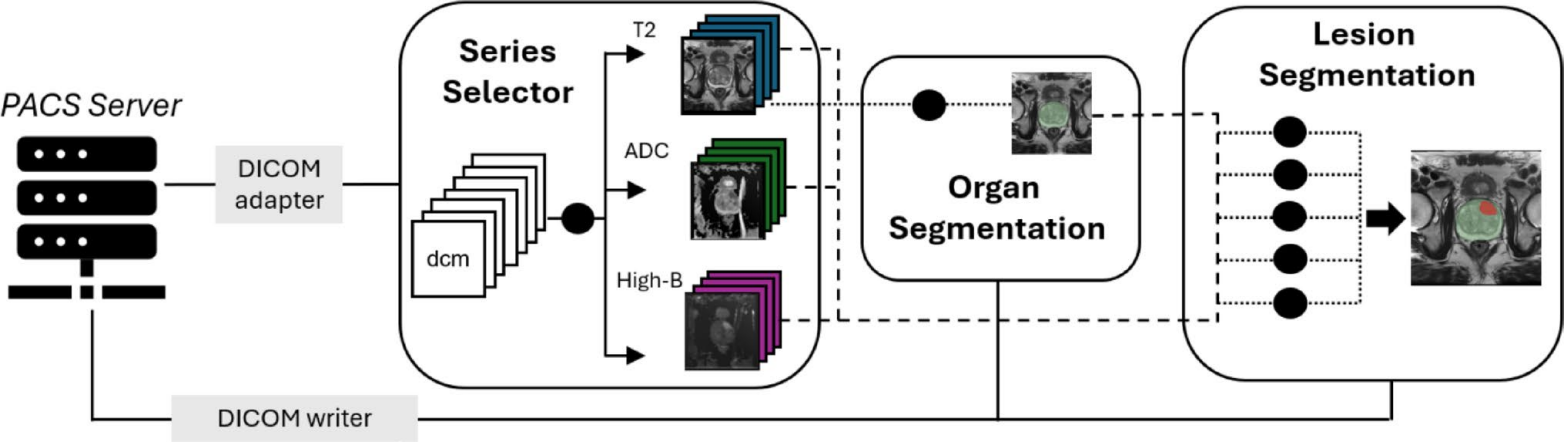
Deployment framework. Data flow from PACS servers is managed by MONAI Deploy Express, with pipeline execution initiated based on application-specific AE titles. Following container execution, the MONAI DICOM Adapter orchestrates the model pipeline. Given the multi-parametric input of the prostate lesion workflow, a Series Selector operator is used to parse DICOM information and sort images into relevant classifications for AI model use. T2W series are then sent to Organ Segmentation Operator for organ-based segmentation. All mpMRI series (T2W, ADC, and high b-value) and organ segmentation mask are resampled to spatial resolution of T2W image and routed to the Lesion Segmentation Operator. This operator runs an ensemble of five lesion segmentation models to produce a final lesion segmentation mask. Results are then routed to custom MONAI DICOM Writer operators, including DICOM series with visual results and RTSS series for downstream clinical workflow. Upon completion of the pipeline container, results are returned from the inference server back to clinical PACS server

A dedicated deployment server was installed within the firewall of our institution’s Radiology department with the following system configuration: Intel^®^ Xeon CPU E5-2667v3 3.2 GHz (32 cores), x84-64bit Linux/GNU, and two T4 NVIDIA GPUs. MONAI was used throughout this work. MONAI Core was used for training the models. MONAI Deploy has several sub-components which were utilized. MONAI Deploy App SDK for building the workflow into a containerized application called a MONAI Application Package (MAP). Finally, MONAI Deploy Express was used for orchestrating the deployment of the MAP and communicating with clinical IT infrastructure such as PACS. Briefly, MONAI Deploy generally uses the concept of “operators” to delineate and describe the functional building blocks of a medical imaging workflow. For the prostate lesion workflow, the main operators include a series selection operator, an organ segmentation operator, and a lesion segmentation operator (Fig. 1).

This work utilized MONAI Deploy Express to package and execute the pipeline (https://github.com/Project-MONAI/monai-deploy/tree/main/deploy/monai-deploy-express). A DICOM Service Class Provider (SCP) is able to receives DICOM images pushed from PACS (Philips/Carestream Vue PACS, v12.2) under Application Entity (AE) Titles specifically associated to each inference pipeline (in this study “CD_ProstateSeg”). Once the DICOMs are received and container executed, the MONAI DICOM Adapter is used to feed images to the series selection operator for parsing the series metadata and route the proper modalities to the correct operators (Fig. 1). While operators select the T2, ADC < and high-b series which are routed to the pipeline, these series still must be sorted and correctly labeled for input to the model. This is done by providing a dictionary of regular expressions (referred to as “Regex”) selection rules that can be continually updated or adjusted based on common DICOM naming conventions for a given institution. We have provided the Regex rules used at our institution as an example in the supplemental material.

Following classification of the series, the selected T2 series is then routed to the organ segmentation operator. The resulting organ segmentation is then combined with the original T2W image along with the ADC maps and high b-value images to create a 4-channel image resampled to match the T2 series spacing. All three MRI sequences are used by the lesion segmentation model, while the organ mask is used to isolate and crop the region of interest (Fig. 1). The probability maps from all models are combined and a threshold is applied to create a final mask. The organ and lesion masks are finally provided to several operators that produce DICOM outputs including a combined mask that is overlaid on the T2, a lesion probability map overlaid on the T2, and individual DICOM RTSTRUCT objects for each mask. These outputs are provided back to the MONAI DICOM Adapter and routed to the PACS or another DICOM endpoint (Fig. 1).

### Phase 1: real-time PACS assessment

**Fig. 2:**
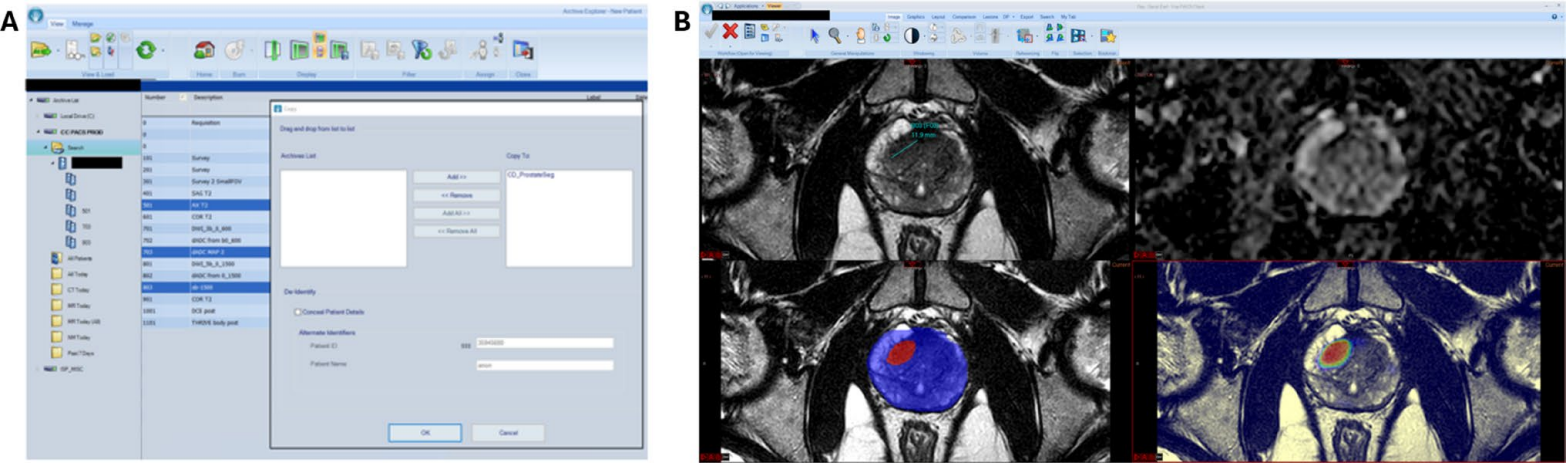
Philips/Carestream Vue PACS system integration. **A** Data flow from PACS servers to inference server, where PACS users are able to push selected DICOM series to pre-specified location for inference. In this example, a pipeline-specific DICOM Copy Location was set up in PACS, labeled here as “CD_ProstateSeg” which identifies the prostate segmentation algorithm to user and is associated with pipeline-specific AE title for inference server when images are sent through this mechanism. **B** Resulting AI output shown as binary mask and probability overlay on T2W images within new DICOM series appended to patient study. In this example, an RTSTRUCT with prostate lesion and organ segmentation boundary points is also received by PACS system (not shown)

Patient selection and pipeline execution were controlled by the PACS user (radiologist), who was required to select the appropriate T2W, ADC, and high b-value series within the mpMRI study before routing to CD_ProstateSeg (Fig. 2A). In this study, diagnostic studies were first prospectively read for clinical purposes by an expert radiologist. Following completion of clinical assessment and reporting, series were selected in PACS and pushed by a radiologist to the clinical deployment server, and results were received back to PACS. The total processing time was recorded for each patient, defined as the container execution time on deployment server. Comparison of AI-produced outputs and radiologist interpretation was completed within the PACS interface (Fig. 2B), where concordance of intra-prostatic lesion detection was evaluated based on the radiologist as reference standard.

### Phase 2: biopsy planning and target definition assessment

Similar to the workflow presented in Phase 1, following completion of clinical assessment and reporting, series were selected in PACS and pushed by a radiologist to the clinical deployment server, and results were received back to PACS. T2W series and AI-produced RTSTRUCT objects were then sent to the UroNav Fusion Biopsy System (Philips). Here, the radiologist has the option to accept/reject AI-based ROIs and modify ROI boundaries for biopsy targeting. Additional lesions identified by the radiologist but not detected by AI were manually contoured. Two biopsy cores of each targeted lesion were obtained under guidance by MRI-TRUS fusion software. Biopsies were performed by urologists and/or radiologists. A single highly experienced genitourinary pathologist reviewed all biopsy specimens and graded each according to ISUP standards [24].

### Statistical analysis

Summary statistics of patient demographics were reported for both phases. Performance metrics were reported separately for each phase as they differed in reference standard.

In Phase 1, association between container runtime and patient features was performed using Pearson correlation coefficient, when appropriate. Performance metrics of AI-based prostate lesion detection was reported on the scan-level and lesion-level compared to the radiologist as reference standard, including Sensitivity, Positive Predictive Value (PPV), Specificity, Negative Predictive Value (NPV), and Accuracy, when appropriate. On the patient-level, a true positive (TP) is any scan with positive intra-prostatic findings by expert radiologist where at least one AI-based lesion was produced. A False Negative (FN) is any scan with positive intra-prostatic findings by expert radiologist where the AI pipeline did not detect any lesions. A False Positive (FP) scan is any scan without notable intra-prostatic findings by the expert radiologist (i.e. PIRADS 1 in non-treated cases or negative for suspicious findings under any other indication) and at least one AI-based lesion was produced. A True Negative (TN) scan is any scan without notable intra-prostatic findings by the expert radiologist where the AI pipeline did not detect any lesions. On the lesion-level, individual lesion-based findings by the expert radiologist were assessed for spatial concordance with the AI-based findings. Here, TP (spatially concordant), FP (AI result with no overlapping radiologist finding), and FN (radiologist finding with no overlapping AI result) were recorded. TN are undefined on the lesion-level.

In Phase 2, Cancer Detection Rates (CDR) was reported on the lesion-level. Each lesion was categorized as positive/negative by AI and positive/negative by the Radiologist, resulting in three possible classifications: AI+/Rad+ (positive in both), AI+/Rad- (identified by AI, not detected in initial clinical read by radiologist but accepted for targeted biopsy), and AI-/Rad+ (not detected by AI, detected by radiologist in initial clinical read and manually contoured for targeted biopsy). On the patient-level, Sensitivity, PPV, Specificity, NPV were reported for the radiologist and AI separately. All calculations were made considering detection of any PCa (Gleason 3 + 3, ISUP Grade Group 1 and above) and clinically significant prostate cancer (csPCa; Gleason 3 + 4, ISUP Grade Group 2 and above).

## Results

### Phase 1 cohort

From 4/1/2024-4/29/2024, 58 study-eligible patients underwent prostate mpMRI for evaluation or suspicion of localized PCa at our institution under the clinical protocol (Table 1). No patient exclusions were made on the basis of prior treatment. Six patients previously underwent treatment for known PCa, including one having completed external-beam radiation therapy, two having undergone brachytherapy, one patient underwent focal ablation of PCa, and two undergoing experimental therapy at our institution (NCT04943536). The expert radiologist identified suspicious intraprostatic findings in 31/58 scans, corresponding to 46 unique intra-prostatic lesions. The summary of findings for lesions eligible to be scored by PIRADSv2.1 interpretation are listed in Table 1.

**Table 1:** Patient demographics and imaging characteristics

Variable	Phase 1	Phase 2
# Patients	58	35
PSA	3.9 (0.3–41)	5.6 (1.8–29.9)
Age	65 (30–82)	69 (47–83)
Prostate volume (cc)	44.5 (18–172)	67 (25–159)
Clinical Indication for MRI		
Untreated / suspicion or known prostate cancer	52	35
s/p treatment for prostate cancer	6	0
Scan-level summary (radiologist)		
Negative for intraprostatic lesion	27	13
Positive for intraprostatic lesion	31	22
Lesion-level (PIRADS)		
1	0	2
2	14	8
3	13	11
4	8	19
5	6	9

Median processing time for real-time AI deployment was 33 s (range 21–50 s), as reported in Table 2. Median prostate volume by radiologist segmentation was 44.5 cc (18–172 cc) and showed a moderate correlation to container runtime (Pearson *r* = 0.46), reflecting the dependence on the lesion-based operator on prostate organ volume input. Agreement between expert radiologist (reference) and AI lesion detection was comparable to prior reports, with 56.5% lesion-based sensitivity of any suspicious intra-prostatic finding and 87.1% scan-level sensitivity (Table 2). Of note, one scan failed to be successfully deployed through the AI pipeline, due to the lack of available high b-value series. This resulted in a failure at the DICOM series selection operator and the pipeline halted without returning a result.

**Table 2:** Phase 1—AI performance characteristics using radiologist as reference standard

Metric	Summary
Successful run	57 (98%)
Container run time (s)	33 (21–50)
Scan-level performance	
Sensitivity	87.1%
Specificity	22.2%
Accuracy	56.9%
PPV	56.3%
NPV	60.0%
Lesion-level performance	
Sensitivity	56.5%
PPV	35.6%

### Phase 2 cohort

From 05/28/24 − 07/09/24, 40 consecutive study-eligible patients underwent prostate mpMRI for evaluation or suspicion of localized PCa at our institution and underwent biopsy planning review, of which 5 patients were excluded from analysis basis of prior treatment (*n* = 3) or a cancelled biopsy session (*n* = 2). Demographics of the 35 patients included in analysis are presented in Table 1. In total, 72 lesions were accepted by the radiologist for biopsy targeting, of which 23 were not initially detected by the radiologist (AI+/Rad-) but were deemed acceptable for biopsy. CDR was highest in concordant lesions (AI+/Rad+) at 82.1% and 42.9% for PCa and csPCa, respectively (Table 3). PCa CDR was higher in AI+/Rad- lesions than AI-/Rad + lesions, though this difference was not observed in csPCa CDR. Within the 23 AI+/Rad- lesions, csPCa was detected in 4 lesions (Table 3) corresponding to four different patients. csPCa was detected *only* in AI + lesion for one patient, reflecting a change in diagnosis with the use of AI. Evaluated further within PIRADS categories, CDR in PIRADS 3 and PIRADS 4 lesions improved for both PCa and csPCa when considering lesions which were positive by AIwas (Table 4). The radiologist and AI had 100% concordance in lesion detection for PIRADS 5 lesions, reflecting no change in CDR.

**Table 3:** Phase 2—CDRs by lesion categorization

Summary	AI+/Rad+ (*n* = 28)	AI+/Rad- (*n* = 23)	AI-/Rad+ (*n* = 21)
# Lesions	28	23	21
Biopsy result			
Benign	5	12	14
3 + 3	11	7	3
3 + 4	9	2	4
4 + 3	1	1	0
4 + 4	1	0	0
4 + 5	1	1	0
PCa CDR	82.1% (23/28)	47.8% (11/23)	33.3% (7/21)
csPCa CDR	42.9% (12/28)	17.4% (4/23)	19.0% (4/21)

**Table 4:** Contribution of AI positivity to CDR in radiologist-determined PIRADS scoring

Rad PIRADS	PCa		csPCa	
	Rad+	AI+/Rad+	Rad+	AI+/Rad+
1	50% (1/2)	–	0% (0/0)	–
2	37.5% (3/8)	66.7% (2/3)	0% (0/8)	0% (0/3)
3	54.5% (6/11)	83.3% (5/6)	36.4% (4/11)	50% (3/6)
4	57.9% (11/19)	70% (7/10)	36.8% (7/19)	40% (4/10)
5	100% (9/9)	100% (9/9)	55.5% (5/9)	55.5% (5/9)

For each PIRADS score, CDR was calculated for the radiologist (considering all Rad + lesions) and for AI + Radiologist (considering only AI + lesions)

## Discussion

Implementation of AI algorithms into clinical practice is almost always limited by lack of integrated systems in clinical departments or barriers in access to commercial/third-party software. Ideal clinical deployment and validation of AI algorithms would allow radiologists to interact with model results directly within clinical PACS viewing environments which they are quite familiar. In this study, we have shared our experience with real-time deployment of an AI-based prostate and lesion segmentation pipeline which was achieved within the clinical radiology environment, utilizing a publicly-available platform (MONAI Deploy Express) on a dedicated inference server.

Accurate detection of suspicious intra-prostatic findings on prostate mpMRI is a critical step in the diagnostic workup and monitoring of PCa, particularly for precise targeting and biopsy sampling of relevant pathological findings [1, 3]. Despite standardized reporting guidelines, mpMRI interpretation is subject to significant inter-reader variability among radiologists [5, 25]. Therefore, detection of intra-prostatic findings on mpMRI has become a popular research topic in the AI field [26, 27]. The inference pipeline performance compared to an expert radiologist in this prospective patient cohort achieved similar results as obtained during original model development [14] and subsequent validation studies. In the largest validation study to date of 658 patients [16], CDR was 44% in AI compared to 49% by an expert Radiologist with biopsy as reference standard. Of note, AI-only lesions were retrospectively mapped to regions of non-targeted biopsy cores and in some cases could not be properly validated as we have done in this study. We have observed that the performance of AI can decrease in low-quality scans from external centers [18]. However, more recently in a study of 144 patients undergoing biopsy at an external academic center, CDR of 86.6% for AI-positive findings compared to 85.7% for radiologist-based assessment in a population of patients with predominantly PIRADS = 4 and PIRADS = 5 lesions [17]. The variation in CDR across these populations reflects known variability in performance of prostate MRI and PIRADS across clinical settings, where the average CDR for PIRADS > = 3 lesions was 35% (95%-CI 27–43%) and ranged from < 20% to > 65% across all centers [5]. Furthermore, we demonstrate these AI-based findings can be useful for ROI-based delineation and improved diagnostic targeting within a commercially available clinical biopsy platform. The model showed excellent concordance with the radiologist for detection of highly suspicious regions (PIRADS 5), and, additionally demonstrated added value for identifying targets not previously identified by the radiologist with 47.8% CDR in ROIs proposed solely by AI and accepted for targeting by the radiologist. While model robustness and clinical impact are outside the scope of this study, PACS-based implementation described here represents an important step towards prospective evaluation of these AI-based solutions in a clinical-trial setting.

This approach was designed to execute deployment and inference with full integration to a clinical PACS system. The seamless integration is important to avoid workflow disruptions on the part of the radiologist and helps deliver results more quickly to the referring clinician and patient. Bidirectional dataflow enabled AI results to be sent directly back to the PACS environment, but the system is flexible to server destinations and other customizations. As we have demonstrated in a pilot cohort of patients, the DICOM-based model results offer flexibility to be ingested by other DICOM locations, such as the commercial biopsy planning software used in this study. Similar reports in literature have highlighted custom solutions for PACS deployment of classical machine learning and deep learning algorithms [28–30]. More recently, deployment of deep-learning based algorithms has been enabled through public platforms such as Clara Deploy [31], though model transportability and lack of public open source code led to the transition of Clara Deploy to MONAI open-source framework efforts. We elected to use this publicly available platform that can be utilized by researchers, clinicians, and industry partners alike, enabling widespread use and multi-center clinical validation [12, 23]. While outside the scope of this research, MONAI framework has the ability to orchestrate multiple AI pipelines at a given time, streamlining the computational resources required for a deployment inference engine.

There are few limitations within this pilot study, which was designed to execute deployment based on the radiology user within PACS, who has advance knowledge of study and patient information for appropriate selection in a clinical trial setting. This user will select the individual series which are relevant for AI processing. Future work includes automated series selection and pipeline execution, either at the MRI scanner console prior to image transfer to PACS environment or automatically within PACS environment before clinical assessment by radiologist. Additionally, as this study aimed to demonstrate feasibility of deployment, only one radiology user deployed and validated these results from the clinical platform in a small population of patients from one center. In Phase 1 of this feasibility study we did not exclude patients on the basis of prior treatment to evaluate whether the algorithm can successfully produce results in challenging settings. This algorithm has been previously validation in two post-treatment settings [20, 21] but is not evaluated for clinical implementation pending larger validation studies. Expanding to additional users will enable a broad assessment of model performance. Finally, while preliminary assessments demonstrate the combination of radiologist and AI achieve the highest cancer detection rates, further investigation on the appropriate selection and use of AI-based biopsy targets is needed.

In conclusion, this short series workflow and real-life clinical implementation confirms feasibility of implementation of a newly developed AI algorithms into clinical PACS environment and sheds light on one promising pathway for translational AI applications in radiology. However, scenarios where AI algorithms are not yet standard of care and should only be used within the context of an IRB approved clinical trial.

**Fig. 3:**
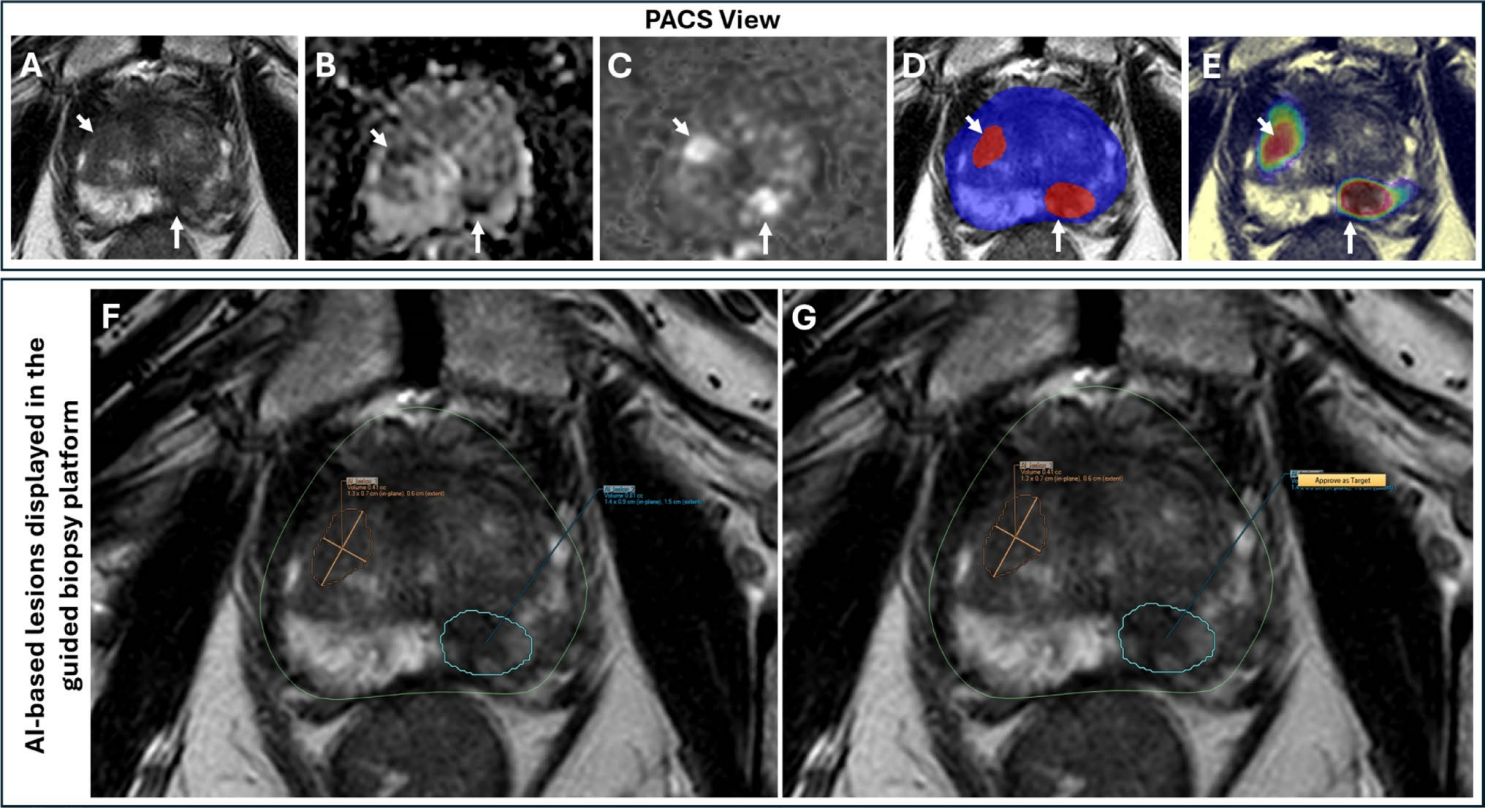
Biopsy planning system integration. 76-year-old patient with a serum PSA of 8.4 ng/ml. Bi-parametric MRI (**A** = T2W MRI, **B** = ADC map, **C** = b1500 DW MRI) demonstrates focal lesions in the left apical peripheral zone (arrow) and right apical anterior transition zone (short arrow). Binary lesion prediction (**D**) and probability (**E**) maps confirm presence of both lesions (long and short arrows). AI-model outputs are presented to users in the biopsy preparation platform (**F**) and the platform allows user to approve or reject the AI-based target lesions (**G**)

## Electronic supplementary material

Below is the link to the electronic supplementary material.

Supplementary Material 1

## Data Availability

The models have been made publicly available for research use https://github.com/Project-MONAI/research-contributions/tree/main/prostate-mri-lesion-seg.
